# Supplementary material for: Multi‐dataset identification of innovative feature genes and molecular mechanisms in keratoconus
Source: J Cell Mol Med. 2024 Sep 19;28(18):e70079. doi: 10.1111/jcmm.70079 (PMC11412914; doi:10.1111/jcmm.70079)
Supplement: Supplementary file 2 — Material S2. [file JCMM-28-e70079-s002.zip › 7.GSEA analysis/4.MYRF/KEGG_SMALL_CELL_LUNG_CANCER.html]

Details for gene set KEGG\_SMALL\_CELL\_LUNG\_CANCER[GSEA]

|  || Dataset | MYRF.group.cls#h\_versus\_l |
| Phenotype | group.cls#h\_versus\_l |
| Upregulated in class | l |
| GeneSet | KEGG\_SMALL\_CELL\_LUNG\_CANCER |
| Enrichment Score (ES) | -0.37414315 |
| Normalized Enrichment Score (NES) | -1.4794623 |
| Nominal p-value | 0.08080808 |
| FDR q-value | 0.2070711 |
| FWER p-Value | 0.787 |
Table: GSEA Results Summary

  

Fig 1: Enrichment plot: KEGG\_SMALL\_CELL\_LUNG\_CANCER      
 Profile of the Running ES Score & Positions of GeneSet Members on the Rank Ordered List

  

| SYMBOL | TITLE | RANK IN GENE LIST | RANK METRIC SCORE | RUNNING ES | CORE ENRICHMENT || 1 | PIK3R5 | na | 661 | 0.528 | 0.0229 | No |
| 2 | CCNE2 | na | 2609 | 0.317 | -0.0080 | No |
| 3 | RARB | na | 3128 | 0.289 | -0.0000 | No |
| 4 | LAMB4 | na | 3997 | 0.254 | -0.0049 | No |
| 5 | APAF1 | na | 5026 | 0.221 | -0.0170 | No |
| 6 | FN1 | na | 6837 | 0.168 | -0.0558 | No |
| 7 | PIK3R1 | na | 7105 | 0.161 | -0.0507 | No |
| 8 | COL4A1 | na | 8213 | 0.133 | -0.0721 | No |
| 9 | PIK3CG | na | 9794 | 0.100 | -0.1098 | No |
| 10 | TRAF3 | na | 10032 | 0.095 | -0.1090 | No |
| 11 | AKT2 | na | 10210 | 0.092 | -0.1068 | No |
| 12 | PTEN | na | 11228 | 0.075 | -0.1303 | No |
| 13 | CCNE1 | na | 11231 | 0.075 | -0.1244 | No |
| 14 | PIK3CA | na | 11255 | 0.074 | -0.1192 | No |
| 15 | E2F3 | na | 11256 | 0.074 | -0.1133 | No |
| 16 | LAMA5 | na | 11263 | 0.074 | -0.1076 | No |
| 17 | XIAP | na | 11781 | 0.066 | -0.1172 | No |
| 18 | PIAS1 | na | 11967 | 0.064 | -0.1174 | No |
| 19 | FHIT | na | 12028 | 0.063 | -0.1142 | No |
| 20 | LAMC3 | na | 12089 | 0.062 | -0.1109 | No |
| 21 | RXRA | na | 12183 | 0.061 | -0.1088 | No |
| 22 | E2F2 | na | 12461 | 0.057 | -0.1123 | No |
| 23 | PIK3R2 | na | 12535 | 0.056 | -0.1099 | No |
| 24 | RB1 | na | 12759 | 0.053 | -0.1121 | No |
| 25 | TP53 | na | 12977 | 0.051 | -0.1144 | No |
| 26 | IKBKB | na | 12994 | 0.050 | -0.1108 | No |
| 27 | TRAF2 | na | 13353 | 0.046 | -0.1175 | No |
| 28 | CDKN1B | na | 14078 | 0.037 | -0.1355 | No |
| 29 | CHUK | na | 14348 | 0.034 | -0.1406 | No |
| 30 | CCND1 | na | 14657 | 0.031 | -0.1470 | No |
| 31 | PIAS4 | na | 16014 | 0.018 | -0.1847 | No |
| 32 | PIK3CB | na | 17199 | 0.007 | -0.2184 | No |
| 33 | MAX | na | 17507 | 0.004 | -0.2269 | No |
| 34 | AKT1 | na | 18101 | -0.001 | -0.2439 | No |
| 35 | BIRC2 | na | 18537 | -0.006 | -0.2560 | No |
| 36 | PIAS3 | na | 19292 | -0.014 | -0.2767 | No |
| 37 | TRAF4 | na | 20246 | -0.025 | -0.3022 | No |
| 38 | PIK3CD | na | 20433 | -0.027 | -0.3054 | No |
| 39 | PIK3R3 | na | 20464 | -0.027 | -0.3042 | No |
| 40 | IKBKG | na | 21687 | -0.042 | -0.3361 | No |
| 41 | E2F1 | na | 21781 | -0.043 | -0.3354 | No |
| 42 | CDKN2B | na | 21823 | -0.044 | -0.3331 | No |
| 43 | LAMB2 | na | 21964 | -0.045 | -0.3335 | No |
| 44 | SKP2 | na | 22430 | -0.051 | -0.3428 | No |
| 45 | CASP9 | na | 23052 | -0.060 | -0.3560 | No |
| 46 | LAMA2 | na | 23159 | -0.062 | -0.3541 | No |
| 47 | TRAF6 | na | 23526 | -0.068 | -0.3593 | No |
| 48 | CYCS | na | 23572 | -0.069 | -0.3551 | No |
| 49 | PTK2 | na | 23650 | -0.070 | -0.3518 | No |
| 50 | ITGA3 | na | 23712 | -0.071 | -0.3479 | No |
| 51 | LAMB1 | na | 24119 | -0.078 | -0.3534 | No |
| 52 | CKS1B | na | 24260 | -0.080 | -0.3511 | No |
| 53 | BIRC3 | na | 24271 | -0.080 | -0.3450 | No |
| 54 | CDK4 | na | 24674 | -0.088 | -0.3496 | No |
| 55 | ITGA2 | na | 25150 | -0.096 | -0.3557 | No |
| 56 | CDK6 | na | 25789 | -0.108 | -0.3655 | Yes |
| 57 | RXRB | na | 25902 | -0.111 | -0.3600 | Yes |
| 58 | ITGA2B | na | 25913 | -0.111 | -0.3514 | Yes |
| 59 | COL4A2 | na | 26047 | -0.114 | -0.3462 | Yes |
| 60 | LAMA4 | na | 26157 | -0.116 | -0.3401 | Yes |
| 61 | PIAS2 | na | 26547 | -0.124 | -0.3415 | Yes |
| 62 | ITGAV | na | 26862 | -0.132 | -0.3401 | Yes |
| 63 | MYC | na | 27707 | -0.153 | -0.3523 | Yes |
| 64 | LAMC1 | na | 27992 | -0.160 | -0.3477 | Yes |
| 65 | LAMC2 | na | 28053 | -0.162 | -0.3366 | Yes |
| 66 | NFKB1 | na | 28241 | -0.168 | -0.3286 | Yes |
| 67 | BCL2L1 | na | 28417 | -0.173 | -0.3199 | Yes |
| 68 | LAMA3 | na | 29367 | -0.202 | -0.3313 | Yes |
| 69 | ITGA6 | na | 29414 | -0.203 | -0.3165 | Yes |
| 70 | CDK2 | na | 29418 | -0.203 | -0.3004 | Yes |
| 71 | COL4A4 | na | 29690 | -0.213 | -0.2913 | Yes |
| 72 | ITGB1 | na | 29873 | -0.219 | -0.2791 | Yes |
| 73 | LAMB3 | na | 30260 | -0.233 | -0.2718 | Yes |
| 74 | RELA | na | 31135 | -0.271 | -0.2755 | Yes |
| 75 | NFKBIA | na | 31525 | -0.292 | -0.2635 | Yes |
| 76 | TRAF5 | na | 32408 | -0.344 | -0.2616 | Yes |
| 77 | PTGS2 | na | 32511 | -0.351 | -0.2366 | Yes |
| 78 | BCL2 | na | 32817 | -0.374 | -0.2157 | Yes |
| 79 | TRAF1 | na | 33097 | -0.397 | -0.1923 | Yes |
| 80 | COL4A6 | na | 33147 | -0.402 | -0.1618 | Yes |
| 81 | LAMA1 | na | 34028 | -0.528 | -0.1452 | Yes |
| 82 | NOS2 | na | 34080 | -0.537 | -0.1039 | Yes |
| 83 | RXRG | na | 34332 | -0.610 | -0.0627 | Yes |
| 84 | AKT3 | na | 34696 | -0.932 | 0.0009 | Yes |
Table: GSEA details [plain text format]

  

Fig 2: KEGG\_SMALL\_CELL\_LUNG\_CANCER      
 Blue-Pink O' Gram in the Space of the Analyzed GeneSet

  

Fig 3: KEGG\_SMALL\_CELL\_LUNG\_CANCER: Random ES distribution      
 Gene set null distribution of ES for **KEGG\_SMALL\_CELL\_LUNG\_CANCER**
